# The m6A regulatory gene YTHDF3 alleviates acute pancreatitis by modulating CD45RA^+^ resting treg cells: A novel immunomodulatory biomarker

**DOI:** 10.1097/MD.0000000000044443

**Published:** 2025-09-12

**Authors:** Huiwen Zhang, Yan Su, Yuedong Hai, Qi Wang

**Affiliations:** a Department of Emergency Surgery, Affiliated Hospital of Inner Mongolia Medical University, Hohhot, People’s Republic of China; b Department of Neurology, Inner Mongolia Autonomous Region People’s Hospital, Hohhot, People’s Republic of China.

**Keywords:** acute pancreatitis, m^6^A methylation, Mendelian randomization, regulatory T cells, YTHDF3

## Abstract

Acute pancreatitis (AP) is an inflammatory disorder with a rising global incidence and substantial morbidity and mortality; however, its molecular pathogenesis remains unclear. Epigenetic regulation by N6-methyladenosine (m6A) RNA modification is increasingly being recognized as a key mechanism in immune and inflammatory diseases. However, the causal role of m6A-related genes and their immunological mediators in AP has not been elucidated. We performed 2-sample Mendelian randomization (MR) analysis to assess the causal relationship between m6A gene expression and AP risk using cis-eQTL data from the eQTLGen consortium and genome-wide association studies summary statistics from 855,309 individuals of European ancestry. Summary-data-based MR (SMR) and heterogeneity in dependent instruments tests were conducted to validate causality and to exclude linkage bias. Furthermore, a 2-step MR mediation analysis was performed using 612 immune cell traits from the Integrative Epidemiology Unit OpenGWAS database to identify downstream immune mediators. Among the 12 m6A genes with valid cis-eQTL instruments, only YTHDF3 demonstrated a significant protective effect against AP (OR = 0.876, 95% CI: 0.795–0.966, *P* = .0078). SMR analysis confirmed this association (b_SMR = −0.176, *P* = .0106) without evidence of heterogeneity (*P*_heterogeneity in dependent instruments = 0.451). Mediation analysis identified CD45RA^+^ resting regulatory T cells as a partial mediator of YTHDF3’s effect (β_a_ = 0.2758; β_b = −0.0297), with an indirect effect accounting for 6.18% of the total causal pathway. Our study identified YTHDF3 as a novel protective gene for AP and suggested that its effect is partially mediated by CD45RA^+^ resting Treg cells. These findings reveal a previously unrecognized m6A-immune axis and offer potential biomarkers and targets for immunomodulatory interventions in AP.

## 1. Introduction

Acute pancreatitis (AP) is a severe acute gastrointestinal disorder with an increasing incidence in recent years. It significantly impairs the patients’ quality of life and can even lead to death. Globally, the incidence of AP has been steadily increasing, affecting approximately 23 to 136 individuals per 100,000 annually.^[1]^ The clinical manifestations typically include severe abdominal pain, nausea, vomiting, and fever. While most cases are self-limiting, approximately 20% develop severe AP, which is characterized by persistent organ failure and high mortality rates exceeding 30%.^[2,3]^ Despite advancements in early resuscitation, nutrition, and supportive care, there are currently no disease-specific interventions that target the underlying molecular drivers of AP, highlighting the urgent need for mechanistic studies and identification of novel therapeutic targets.

Recent evidence indicates that dysregulated immune responses and inflammation are central to AP pathogenesis.^[4]^ However, although pro-inflammatory pathways have been extensively studied, the identification of endogenous protective genes and immune-regulatory pathways remains limited. In this context, posttranscriptional RNA modification by N6-methyladenosine (m6A) has emerged as a crucial regulator of gene expression, affecting mRNA splicing, stability, translation, and degradation.^[5]^ Among the key m6A “reader” proteins, YTH N6-methyladenosine RNA-binding protein 3 (YTHDF3) has been shown to influences translation efficiency and mRNA decay.^[6,7]^ However, its role in inflammation and pancreatic diseases remains largely unexplored.

To systematically investigate whether m6A-modified genes, such as YTHDF3, are causally involved in AP, we integrated cis-expression quantitative trait loci (cis-eQTL) data from the eQTLGen consortium with genome-wide association studies (GWAS) on AP involving over 855,000 European participants. Using a 2-sample Mendelian randomization (MR) framework, we screened 12 m6A regulatory genes and identified YTHDF3 as a significant protective factor (OR = 0.876, *P* = .0078). These findings were corroborated using summary-data-based MR (SMR) and heterogeneity in dependent instruments (HEIDI) heterogeneity tests, which confirmed the causal effect of YTHDF3 expression on reduced AP risk and excluded the possibility of spurious associations due to linkage disequilibrium (LD).

To elucidate the immunological pathway mediating this effect, we conducted MR-based mediation analysis using immune cell trait data from the Integrative Epidemiology Unit OpenGWAS project. Our analysis identified CD45RA^+^ resting regulatory T cells (Tregs) as a downstream effector mediating the protective effect of YTHDF3. Specifically, YTHDF3 expression was positively associated with CD45RA^+^ Tregs (β_a_ = 0.2758), which in turn showed a negative association with AP risk (β_b = −0.0297), suggesting a suppressive, immunomodulatory role of this Treg subset. Although the mediation proportion was modest (6.18%), this is the first evidence linking m6A gene expression to Treg-mediated immune protection in AP.

Taken together, our study is the first to reveal a YTHDF3–CD45RA^+^ Treg axis that may modulate AP susceptibility, bridging RNA epigenetics with immune regulation. By applying a robust analytical pipeline including eQTL mapping, MR, SMR, and mediation models, our findings not only enhance the understanding of AP pathophysiology, but also highlight YTHDF3 as a promising biomarker and potential target for future immune-based interventions.

## 2. Materials and methods

### 2.1. Data source

In this study, 19 N6-methyladenosine (m6A) regulatory genes were included for analysis,^[8–11]^ with the complete gene list provided in Table S1, Supplemental Digital Content, https://links.lww.com/MD/P921. Expression quantitative trait loci (eQTL) data for all genes were obtained from the eQTLGen Consortium database (https://www.eqtlgen.org/cis-eqtls.html).^[12]^ All selected eQTLs were cis-acting (cis-eQTLs) and derived from whole blood samples. To identify suitable instrumental variables (IVs) for MR analysis, single nucleotide polymorphisms (SNPs) were filtered using the following criteria: *P*-value <5 × 10^−8^, LD clumping with *r*² <0.1, and a clumping window of 10,000 kb. After applying these thresholds, 15,695 genes with cis-eQTL data were retained. The intersection between these genes and the predefined 19 m6A genes yielded 12 m6A genes with valid cis-eQTL instruments for downstream MR analysis. Summary statistics for AP were obtained from the GWAS Catalog database (https://www.ebi.ac.uk/gwas/). This GWAS included 855,309 individuals of European ancestry, comprising 10,630 cases and 844,679 controls.

### 2.2. Two-sample Mendelian randomization analysis

Two-sample MR analyses were performed using the TwoSampleMR package in R. The cis-eQTL data for m6A regulatory genes were used as exposures, and AP was considered as the outcome. To minimize bias from weak instruments, SNPs with *F*-statistics <10 were excluded prior to the analysis. The inverse variance weighted (IVW) method was employed as the primary analytical approach. Exposures without valid IVW estimates were excluded from further MR analysis. We applied the MR-Egger intercept test to assess potential horizontal pleiotropy. Heterogeneity among the IVs was evaluated using Cochran *Q* statistic. Furthermore, a leave-one-out sensitivity analysis was performed to examine the robustness and stability of MR estimates.

### 2.3. Summary-data-based Mendelian randomization analysis

SMR analysis was conducted to evaluate pleiotropic associations between gene expression levels and complex traits of interest using summary-level data from both GWAS and expression quantitative trait loci (eQTL) studies. The HEIDI test was applied to assess the presence of horizontal pleiotropy within colocalized signals. The null hypothesis of the HEIDI test posits the absence of horizontal pleiotropy, which refers to the scenario in which a single genetic variant affects multiple traits through pathways independent of the exposure under investigation.^[13]^ Together, the SMR and HEIDI tests enable the distinction between causal effects mediated by gene/protein expression and associations arising through alternative biological mechanisms. SMR analysis was performed using SMR software (version 1.3.1), which was downloaded from the official SMR website (https://yanglab.westlake.edu.cn/software/smr/#Overview). The default parameters provided by the SMR tool were used for all analyses.

### 2.4. Exploration of gene function mechanism via mediation analysis

To investigate whether YTHDF3 exerts its effect on AP through modulation of immune cells, we performed a 2-step MR mediation analysis. Summary statistics for immune cell traits were obtained from the Integrative Epidemiology Unit OpenGWAS project (https://gwas.mrcieu.ac.uk/)(ID: ebi-a-GCST90001391–ebi-a-GCST90002121),^[14]^ specifically from datasets with IDs ranging from ebi-a-GCST90001391 to ebi-a-GCST90002121. IVs (SNPs) were selected based on the following thresholds: *P*-value <5 × 10^−8^, clumping window = 10,000 kb, and LD *r*² <0.1, resulting in 612 immune cells traits with valid instruments. First, we estimated the total effect of YTHDF3 expression on AP, denoted as β_c_. Second, we quantified the effect of YTHDF3 eQTLs on each immune cell trait (β_a_) and the effect of each immune cell trait on AP (β_b_). The mediated (indirect) effect was calculated as β_a_ × β_b_= β_ab_ and the proportion mediated was derived as (β_ab_/ β_c_) × 100% = β_c′_.

### 2.5. Statistical analysis

All statistical analyses were performed using R software (version 4.4.2), which is an open-source and freely available platform. Statistical significance was defined as a 2-sided *P*-value of <.05.

## 3. Results

### 3.1. Identification of m6A genes for Mendelian randomization analysis

**Figure 1.**
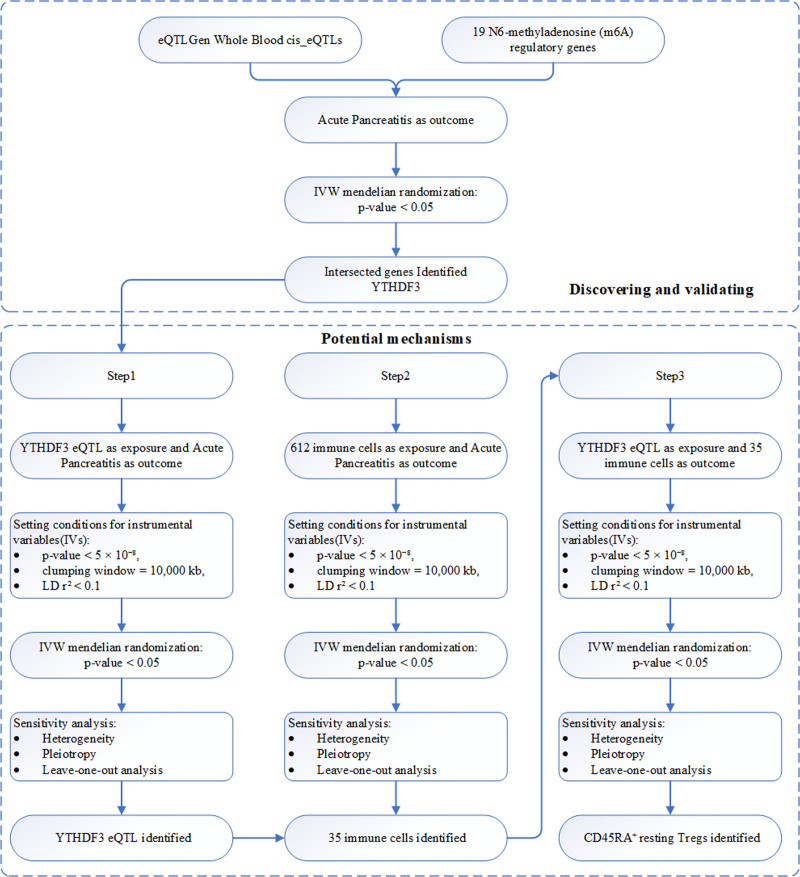
The detailed study design of this study.

**Figure 2.**
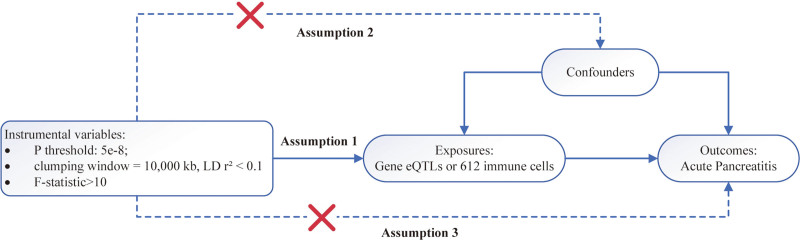
Schematic illustration of the 3 core assumptions of MR. This diagram outlines the fundamental assumptions required for valid MR analysis. Assumption 1 (relevance): The selected IVs must be strongly associated with the exposure (gene expression or immune cell traits). SNPs were clumped using a *P*-value threshold of 5 × 10^−8^, LD *r*² <0.1, and a clumping window of 10,000 kb; only instruments with *F*-statistics > 10 were retained. Assumption 2 (independence): the genetic instruments must be independent of any confounders that influence both the exposure and the outcome (acute pancreatitis). Assumption 3 (exclusion restriction): the instruments must affect the outcome solely through the exposure and not through any alternative pathways (i.e., no horizontal pleiotropy). The red crosses indicate the pathways that are assumed to be absent in a valid MR framework. IVs = instrumental variables, LD = linkage disequilibrium, MR = Mendelian randomization, SNPs = single nucleotide polymorphisms.

**Figure 3.**
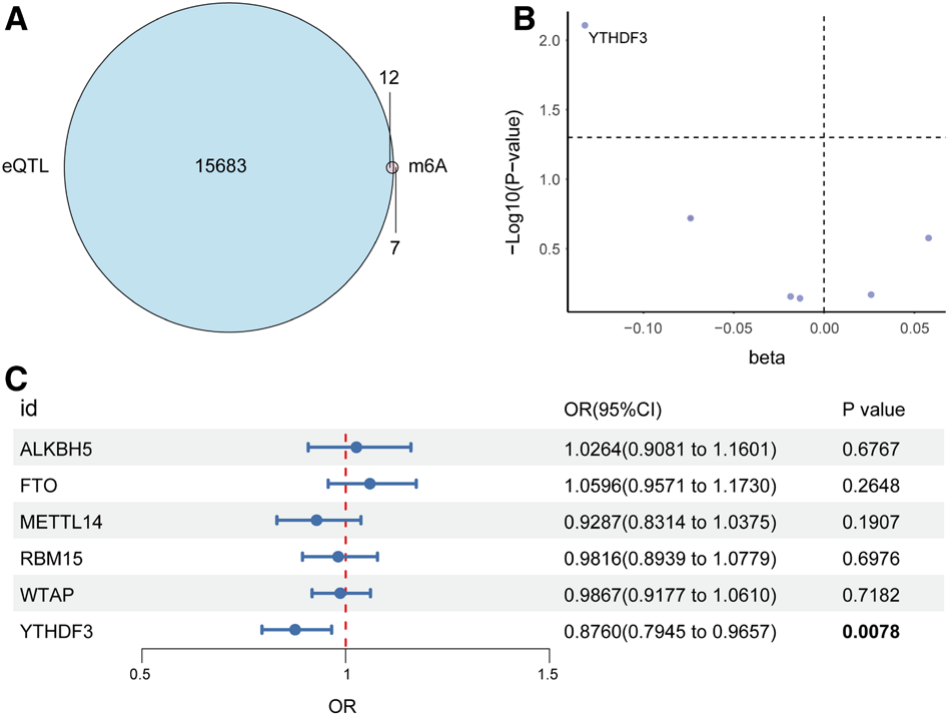
Identification of YTHDF3 as a protective m^6^A regulator gene for acute pancreatitis through MR analysis. (A) Venn diagram showing the overlap between 15,695 genes with valid cis-eQTLs (from the eQTLGen consortium) and a predefined set of 19 m^6^A regulatory genes. A total of 12 m^6^A genes with available cis-eQTL instruments were identified and selected for downstream Mendelian randomization (MR) analysis. (B) Volcano plot displaying MR effect sizes (beta) and significance (−log_10_ *P*-values) of m^6^A genes on AP risk. YTHDF3 was the only gene located in the statistically significant region, with both a negative beta and a *P*-value <.01. (C) Forest plot summarizing the IVW MR estimates for 6 m^6^A genes. ORs, 95% CIs, and *P*-values are presented. Only YTHDF3 showed a statistically significant protective effect against AP (OR = 0.8760, 95% CI: 0.7945–0.9657, *P* = .0078). AP = acute pancreatitis, CIs = confidence intervals, cis-eQTL = cis-expression quantitative trait loci, IVW = inverse variance weighted, MR = Mendelian randomization, ORs = odds ratios, YTHDF3 = YTH N6-methyladenosine RNA-binding protein 3.

The detailed study design is shown in Figure 1. All of our analyses followed the 3 basic assumptions of MR, and their illustrative diagrams are shown in Figure 2. Based on predefined filtering criteria, we identified 15,695 genes with valid cis-eQTLs in the whole blood. Intersecting this list with the 19 curated m6A regulatory genes yielded 12 m6A genes possessing eligible genetic instruments (Fig. 3A). These 12 genes were selected for subsequent MR analyses to explore their potential causal associations with AP.

### 3.2. Mendelian randomization analysis of 12 m6A genes

To investigate whether m6A-related gene expression is causally associated with AP, we performed a 2-sample MR analysis for a total of 12 m6A genes using the IVW method. As shown in Figure 3C, most genes had odds ratios close to 1.0, with wide 95% confidence intervals crossing the null line, and nonsignificant *P*-values (all *P* > .1). However, YTHDF3 exhibited a significant protective effect against AP. IVW analysis showed that higher YTHDF3 expression was associated with reduced AP risk (β = −0.1324, OR = 0.876, 95% CI: 0.795–0.966, *P* = .0078), suggesting a potential causal role. This result was further supported by the volcano plot (Fig. 3B), which plotted the effect size (β) on the x-axis and statistical significance (−log10 *P*-value) on the y-axis. YTHDF3 was the only gene located in the significant region (lower β, higher − log10 p), further confirming its robust inverse association with the disease risk.

To validate the robustness of this association, we conducted additional MR sensitivity analyses by using various complementary methods. As shown in Figure S1, Supplemental Digital Content, https://links.lww.com/MD/P920, the forest plot demonstrated consistent negative effect estimates across SNPs using both IVW and MR-Egger methods. A funnel plot revealed a symmetric distribution of SNPs around the causal estimate lines, suggesting minimal directional pleiotropy. The leave-one-out analysis confirmed that the association was not driven by any single SNP, whereas the scatter plot showed consistent negative slopes across multiple MR models (IVW, MR-Egger, weighted median, and weighted mode), further supporting the stability and directionality of the causal effect (Table S2, Supplemental Digital Content, https://links.lww.com/MD/P921). Taken together, these findings suggest that YTHDF3 may play a protective role in AP, and was therefore selected for downstream mechanistic mediation analysis.

### 3.3. *SMR analysis validates the causal effect of YTHDF3 on acute* pancreatitis

To further validate the MR results and rule out potential LD confounding, we performed SMR analysis for YTHDF3, using rs6993229 as the top instrumental SNP. SMR analysis showed a significant association between YTHDF3 expression and AP risk, with an effect estimate of b_SMR = −0.176, standard error = 0.0689, and p_SMR = 0.0106, consistent with the direction and magnitude of the previous IVW results. This suggests that higher genetically predicted YTHDF3 expression is causally linked to a lower AP risk.

Importantly, the HEIDI test showed no evidence of heterogeneity (p_HEIDI = 0.451, nsnp = 20), supporting the assumption that the observed effect was not due to linkage, but instead likely reflects a shared causal variant. Detailed analysis results are shown in Table S3, Supplemental Digital Content, https://links.lww.com/MD/P921. These findings strengthen the causal inference of YTHDF3 in the pathogenesis of AP and justify its inclusion in downstream mediation analyses.

### 3.4. *Mediation analysis of YTHDF3 and acute pancreatitis via CD45RA*^*+*^ *resting treg*

To elucidate the potential immunological mechanisms through which YTHDF3 eQTL influence the risk of AP, we implemented a 3-step analytical strategy, as illustrated in Figure 1. Step 1 involved using the YTHDF3 cis-eQTL as the exposure and AP as the outcome in a 2-sample MR analysis, which established a significant protective causal association. Step 2 utilized a panel of 612 immune cell traits as exposures and AP as the outcome, identifying 35 immune cell subtypes with significant causal effects on susceptibility to AP. Step 3 assessed whether YTHDF3 eQTL causally affected the levels or proportions of these 35 immune cell types. Among them, CD45RA^+^ resting regulatory T cells were identified as the most likely downstream immune mediators, suggesting a potential immunoregulatory mechanism by which YTHDF3 confers protection against AP.

To explore the immunological mechanism through which YTHDF3 influences the risk of AP, we conducted a 2-step MR mediation analysis, focusing on CD45RA^+^ resting regulatory T cells (Tregs) as the intermediate. Initial MR analysis demonstrated that increased YTHDF3 expression, driven by its cis-eQTL, was significantly associated with elevated levels of CD45RA^+^ resting Tregs (Path a: β_a_ = 0.2758, *P* = 1.47 × 10^−5^). Sensitivity analyses including pleiotropy and heterogeneity testing confirmed the robustness of this association (Fig. S2, Supplemental Digital Content, https://links.lww.com/MD/P920, and Table. S4, Supplemental Digital Content, https://links.lww.com/MD/P921).

In the second step, CD45RA^+^ resting Tregs were causally protective against AP (Path b: β = −0.0297, OR = 0.885, 95% CI: 0.798–0.981, *P* = .021), as shown in Figure S3, Supplemental Digital Content, https://links.lww.com/MD/P920, and Table. S5, Supplemental Digital Content, https://links.lww.com/MD/P921. This suggests a suppressive immunoregulatory role for this Treg subset in AP pathogenesis. By combining both steps, we estimated the total effect of YTHDF3 on AP (Path c) to be βc = −0.1324, with a direct effect (Path c′) of βc′ = −0.1242 and an indirect (mediated) effect through CD45RA^+^ resting Tregs of βab = −0.0082. This mediated path accounted for approximately 6.18% of the total effect, indicating partial but biologically meaningful immunological mediation.

Taken together, these findings suggest that YTHDF3 may exert its protective effect on acute, partly by enhancing the suppressive function of CD45RA^+^ resting Treg cells, providing novel mechanistic insights into its role in immune regulation.

## 4. Discussion

AP is an acute gastrointestinal disorder characterized by premature activation of pancreatic enzymes and subsequent inflammatory responses in the pancreatic tissue. While mild forms of AP are often self-limiting, severe cases can rapidly progress to systemic inflammatory response syndrome and multiple organ dysfunction syndrome, leading to high morbidity and mortality.^[2,3]^ Currently, clinical management is largely limited to supportive care with no effective targeted therapeutic interventions available. Therefore, there is an urgent need to elucidate the molecular mechanisms underlying AP pathogenesis and to identify novel early warning biomarkers and immunomodulatory targets. In recent years, epigenetic mechanisms, particularly RNA modifications, have attracted increasing attention in the context of inflammatory diseases. Among these, N6-methyladenosine (m6A) methylation, a dynamic and reversible RNA modification, has emerged as a critical regulator of gene expression, and may play a pivotal role in the initiation and progression of AP.

The functional potential of the N6-methyladenosine (m6A) modification in inflammation and immunity has garnered growing scientific interest. As a predominant and dynamic form of RNA modification, m6A plays critical roles in various biological processesincluding cell proliferation, differentiation, and apoptosis, which are intimately involved in immune responses. Emerging evidence suggests that m6A contributes significantly to the pathogenesis of several inflammatory disorders, such as rheumatoid arthritis, inflammatory bowel disease, and non-small cell lung cancer.^[15–17]^ These studies revealed that m6A modulates immune homeostasis by regulating immune cell activation, proliferation, and cytokine expression. Although the role of m6A in AP, a prototypical inflammatory disease, remains largely unexplored, preliminary evidence indicates that m6A may influence both the local pancreatic immune microenvironment and the systemic inflammatory response. Recent studies have demonstrated that m6A modifications can affect macrophage polarization and their pro- or anti-inflammatory functions, thereby shaping the course of inflammatory diseases.^[18,19]^ For instance, m6A-regulated signaling pathways are closely linked to macrophage-mediated inflammation and may modulate the pathophysiology of pancreatitis by influencing macrophage activity.^[20,21]^ Moreover, m6A alterations may affect cellular metabolism and lipid homeostasis, further amplifying the inflammatory cascade. Under inflammatory conditions, m6A modifications may fine-tune the expression of key immune and metabolic genes, thereby affecting both the function and crosstalk of immune cells within the inflamed pancreas.^[22,23]^ Therefore, dissecting the molecular mechanisms by which m6A contributes to the pathogenesis of AP may not only enhance our understanding of its role in immune regulation but also provide novel therapeutic targets. Despite the limited current knowledge, accumulating preliminary findings support a potentially pivotal role for m6A in orchestrating immune and inflammatory responses during AP. Future studies should aim to delineate how m6A remodeling shapes both the local and systemic immune landscapes in AP, thereby offering new avenues for targeted intervention.

In this study, we first performed systematic MR analysis using eQTL data for 12 canonical m6A regulatory genes. Notably, elevated YTHDF3 expression was significantly associated with a reduced risk of AP (β = −0.1324, OR = 0.876, *P* = .0078). This causal relationship was further supported by SMR analysis, and no evidence of heterogeneity was detected by the HEIDI test (*P* = .451), effectively excluding the potential confounding effect of LD. YTHDF3 encodes a cytoplasmic RNA-binding protein that plays a pivotal role in determining mRNA fate. By recognizing m6A-modified transcripts, YTHDF3 facilitates either translational initiation or accelerated mRNA decay, thereby modulating a wide range of cellular processes including differentiation, proliferation, immune responses, and stress adaptation. Previous studies have implicated YTHDF3 in diverse diseases. In cancer biology, YTHDF3 has been linked to tumor metastasis and immune evasion. For instance, it promotes the progression and metastasis of triple-negative breast cancer by stabilizing ZEB1 mRNA^[24]^ and enhances hepatocellular carcinoma progression by stabilizing EGFR mRNA and activating the STAT3 signaling pathway.^[25]^ In non-small cell lung cancer, high expression of YTHDF3 correlates with immune escape mechanisms, possibly via modulation of PD-L1 transcriptional stability.^[26]^ In immune and inflammatory diseases such as rheumatoid arthritis, inflammatory bowel disease, pulmonary injury, and infectious diseases, YTHDF3 regulates inflammatory cytokine expression, limits immune overactivation, and preserves tissue homeostasis.^[27,28]^ For example, in autoimmune uveitis, YTHDF3 downregulation is associated with enhanced microglial inflammatory responses, suggesting its role in modulating microglial phenotypes and immunological functions.^[29]^ The involvement of YTHDF3 in Crohn is noteworthy. m6A methylation has been implicated in the pathogenesis of Crohn disease, and YTHDF3, as a key m6A “reader,” participates in the posttranscriptional regulation of inflammation-related genes.^[30]^ In murine models of pneumonia and colitis, elevated YTHDF3 expression has been linked to the suppression of excessive inflammation through the degradation of pro-inflammatory mRNAs.^[31,32]^ In addition to peripheral inflammation, YTHDF3 also plays a critical role in neuroinflammation. In experimental autoimmune uveitis models, upregulation of YTHDF3 is associated with microglial inflammatory activation, suggesting that YTHDF3 may modulate neuroinflammation by influencing microglial phenotype switching.^[33]^ However, to date, the role of YTHDF3 in AP has not been reported. Given that AP is an inflammation-driven, self-limiting disorder characterized by cytokine storms, immune dysregulation, and tissue necrosis, we hypothesized that YTHDF3 exerts protective effects through similar immunoregulatory mechanisms. Therefore, this study aimed to test this hypothesis by employing systematic genetic causal inference strategies.

In the mediation analysis, we proposed for the first time that the m6A reader protein YTHDF3 may influence the risk of AP via immunological pathways, with CD45RA^+^ resting regulatory T-cells (Tregs) acting as partial mediators. Although the proportion mediated was modest at 6.18%, this result revealed a specific regulatory axis between the m6A-modifying genes and immune cell subpopulations. Tregs play a fundamental role in immune regulation, including suppression of effector T cell activity and promotion of immune tolerance in other immune cell subsets.^[34]^ This aligns well with the known role of YTHDF3 in modulating the immune microenvironment, particularly in tumors, where YTHDF3 expression has been associated with immune evasion mechanisms. For instance, several studies have reported a correlation between high YTHDF3 expression and immune cell infiltration, including CD8 + T cells and Tregs, in the tumor microenvironment.^[35,36]^ CD45RA^+^ resting Tregs represent a subset of Treg cells in a naïve or resting state, and are characterized by strong immunosuppressive and tissue-protective functions during the early stages of inflammation. Specifically, these cells suppress effector T-cell activity through multiple mechanisms, including the secretion of immunosuppressive cytokines (e.g., IL-10 and TGF-β) and cell-cell contact–dependent inhibition of T-cell proliferation and activation. They are particularly crucial in the early inflammatory phase, where they help dampen excessive immune responses and facilitate tissue repair and regeneration.^[37]^ The importance of this subset of Tregs has been highlighted in various physiological and pathological contexts. For instance, in neonates, the frequency and functionality of Treg cells may influence immune tolerance to environmental antigens. A study on neonatal immunity demonstrated that the proportion of CD45RA^+^ resting Tregs among CD4 + FoxP3^+^ T cells significantly increased shortly after birth, suggesting a role in protecting against excessive immune activation.^[38]^ In liver transplantation, the frequency and functionality of CD45RA^+^ Tregs have been linked to long-term immune tolerance, underscoring their relevance to graft acceptance.^[39]^ Similarly, in systemic lupus erythematosus, impaired differentiation of Tregs is associated with reduced immunoregulatory capacity and increased disease activity.^[40]^ Importantly, resting Tregs possess the capacity to rapidly transition to an activated state upon antigen encounter, thereby enhancing their suppressive functions.^[41,42]^ In the early phase of AP, CD45RA^+^ resting Tregs may swiftly convert into activated Tregs, playing a critical role in preventing pancreatic tissue injury and promoting the resolution of inflammation.^[43–45]^ Our study demonstrated that elevated YTHDF3 expression is associated with an increased proportion of this Treg subset, indicating its potential to promote immune homeostasis and exert anti-inflammatory effects, thereby alleviating the development of AP. These findings are consistent with previous reports that underscore the importance of Tregs in immune regulation. However, it should be noted that the protective effect of YTHDF3 on AP is not solely mediated by the Treg pathway. Additional mechanisms may involve the regulation of pro-inflammatory mRNA stability, T-cell function, or other m6A-dependent pathways. Further experimental studies using animal models and clinical tissue samples are required to validate the function and generalizability of this regulatory axis.

This study had several limitations that should be acknowledged. First, although our analysis was based on a large sample size, the data were limited to individuals of European ancestry, which may restrict the generalizability of the findings and overlook the potential genetic and environmental heterogeneity across other ethnic populations. Second, this study lacked experimental (wet-lab) validation, preventing direct observation of the biological function and mechanistic role of YTHDF3 in AP. Consequently, the molecular pathways through which YTHDF3 exerts its regulatory effects remain to be elucidated. Third, despite the use of MR to infer causality, residual confounding factors could not be entirely ruled out. Unmeasured factors, such as lifestyle, dietary habits, and other genomic variants, may still influence the observed associations and introduce bias in the causal estimates. Therefore, future studies should aim to validate these findings in multi-ethnic populations and incorporate in vivo and in vitro functional experiments to enhance the reliability and translational potential of these results.

## 5. Conclusions

In summary, this study revealed a potential protective role of YTHDF3 in AP and established a causal relationship through both 2-sample MR and SMR analysis. These findings identified YTHDF3 as a novel biomarker and therapeutic target for the prevention and treatment of AP, particularly in the context of immune regulation. Future research should focus on the functional validation and mechanistic investigation of YTHDF3 to provide more effective clinical intervention strategies, with the ultimate goal of improving the prognosis of patients with AP.

## Author contributions

**Conceptualization:** Yuedong Hai.

**Data curation:** Yuedong Hai.

**Formal analysis:** Huiwen Zhang, Yuedong Hai, Qi Wang.

**Funding acquisition:** Qi Wang.

**Investigation:** Huiwen Zhang, Qi Wang.

**Methodology:** Huiwen Zhang, Yan Su.

**Project administration:** Yan Su.

**Resources:** Yan Su.

**Software:** Yan Su.

**Supervision:** Huiwen Zhang.

**Validation:** Huiwen Zhang.

**Visualization:** Huiwen Zhang.

**Writing – original draft:** Huiwen Zhang.

**Writing – review & editing:** Huiwen Zhang.

## Supplementary Material




