# Supplementary material for: The m6A regulatory gene YTHDF3 alleviates acute pancreatitis by modulating CD45RA+ resting treg cells: A novel immunomodulatory biomarker
Source: Medicine (Baltimore). 2025 Sep 12;104(37):e44443. doi: 10.1097/MD.0000000000044443 (PMC12440514; doi:10.1097/MD.0000000000044443)

Supplementary Figure 1. Sensitivity analyses of the causal effect of YTHDF3 expression on acute pancreatitis using multiple MR approaches.
(A) Forest plot showing the SNP-specific causal estimates and overall effects based on inverse variance weighted (IVW) and MR-Egger methods.
(B) Funnel plot assessing potential directional pleiotropy. The symmetrical distribution of SNPs suggests minimal pleiotropic bias.
(C) Leave-one-out analysis indicating that no single SNP drives the overall association between YTHDF3 and acute pancreatitis.
(D) Scatter plot of SNP effect sizes on exposure (YTHDF3 expression) versus outcome (acute pancreatitis), with regression lines from five MR methods showing consistent negative slopes.


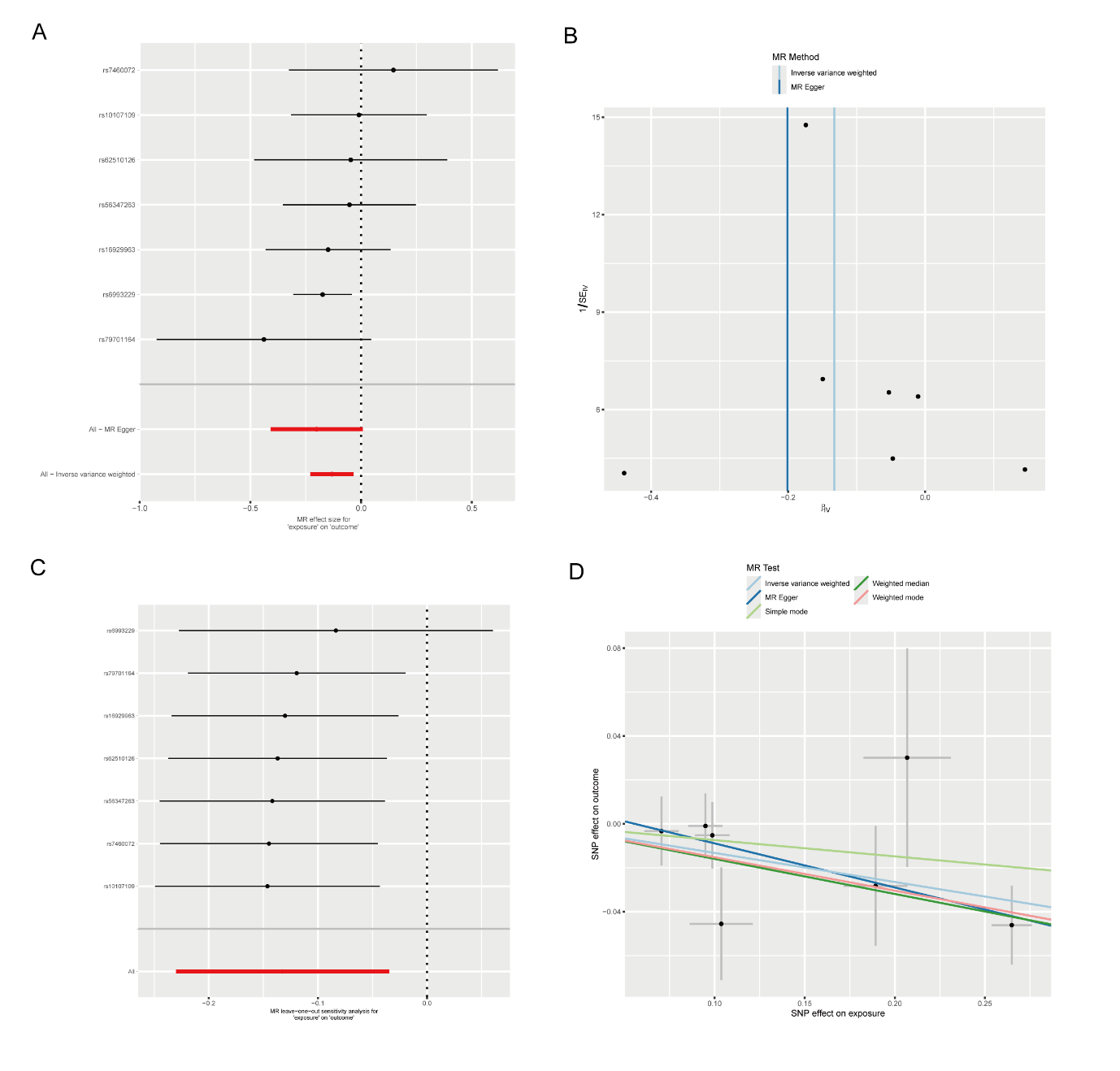


Supplementary Figure 2. Mendelian randomization analyses assessing the causal effect of YTHDF3 expression on CD45RA⁺ resting regulatory T cells.
(A) Forest plot displaying SNP-specific and overall causal estimates from inverse variance weighted (IVW) and MR-Egger methods.
(B) Funnel plot evaluating the presence of directional pleiotropy. The symmetric distribution of SNPs suggests no substantial pleiotropic bias.
(C) Leave-one-out sensitivity analysis confirming that the association is not driven by any single instrumental SNP.
(D) Scatter plot of SNP effects on YTHDF3 expression (exposure) versus effects on CD45RA⁺ resting Tregs (outcome), with regression lines from five MR methods. The consistent positive slopes support a causal effect of YTHDF3 on increased Treg abundance.


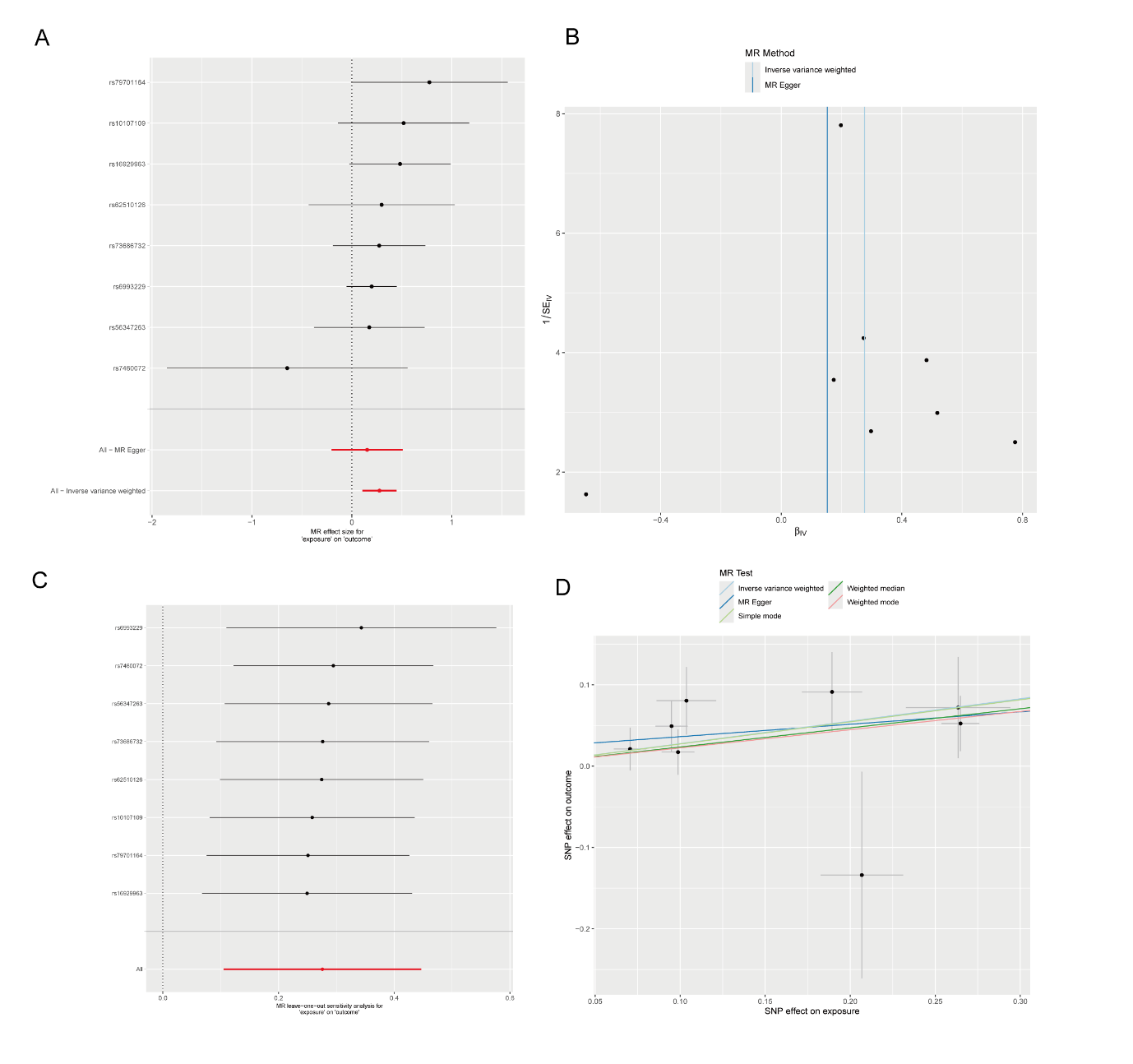


Supplementary Figure 3. Mendelian randomization analyses assessing the causal effect of CD45RA⁺ resting regulatory T cells on the risk of acute pancreatitis.
(A) Forest plot showing SNP-specific and overall MR estimates using inverse variance weighted (IVW) and MR-Egger methods.
(B) Funnel plot evaluating horizontal pleiotropy. The symmetrical distribution of instrumental variables suggests minimal directional bias.
(C) Leave-one-out sensitivity analysis indicating that no single SNP disproportionately influenced the overall causal estimate.
(D) Scatter plot of SNP effects on CD45RA⁺ resting Treg cells (exposure) and acute pancreatitis (outcome), with regression lines from multiple MR methods. The consistent negative slopes support a protective role of CD45RA⁺ resting Tregs in acute pancreatitis pathogenesis.


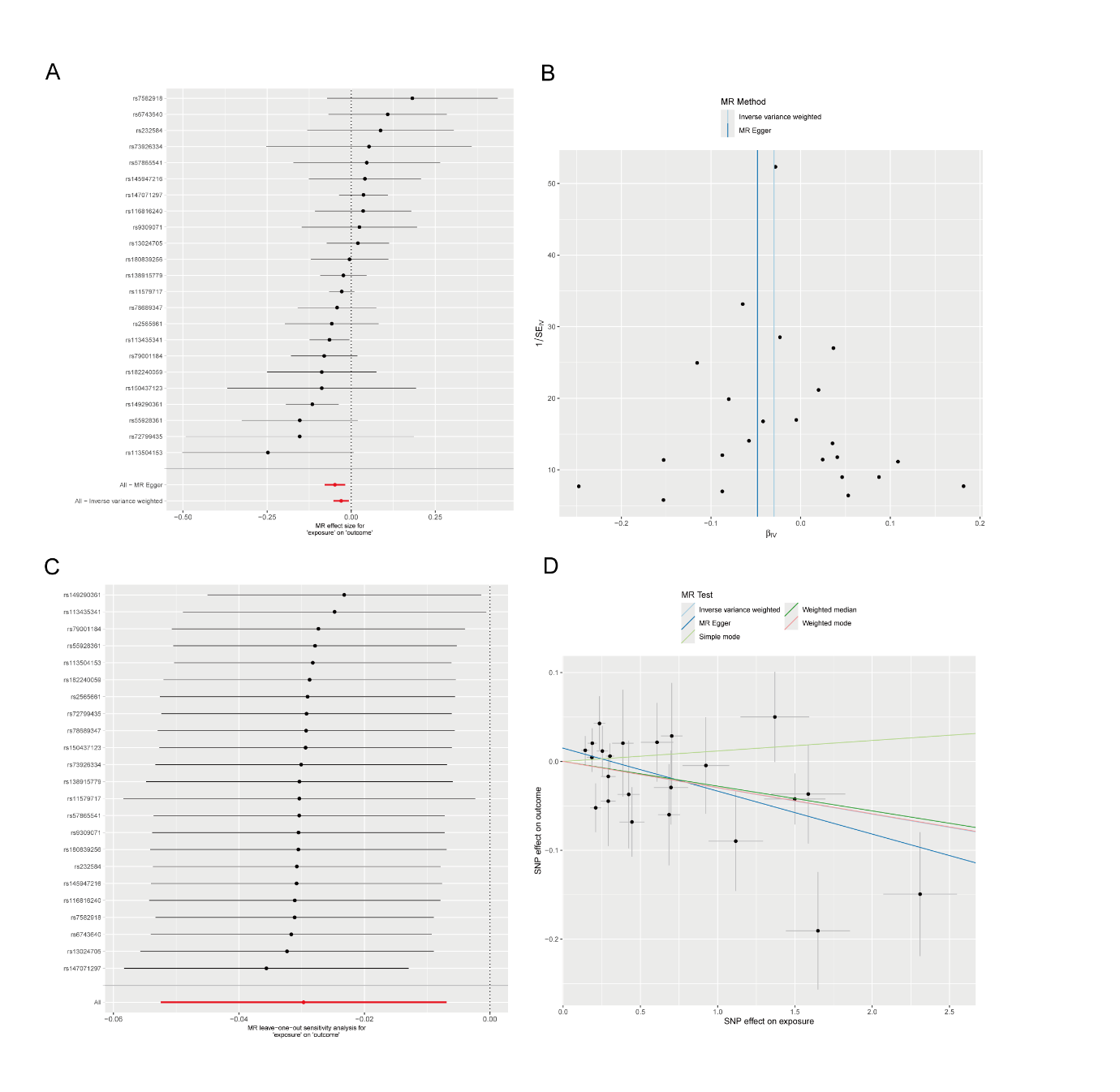

Supplement: Supplementary file 2 [file medi-104-e44443-s002.docx]
